# Measuring handgrip strength in school children: inter-instrument reliability between Takei and Jamar

**DOI:** 10.1038/s41598-024-51368-1

**Published:** 2024-01-11

**Authors:** Nebojša Trajković, Doroteja Rančić, Tamara Ilić, Romina Herodek, Georgiy Korobeynikov, Damir Pekas

**Affiliations:** 1 Faculty of Sport and Physical Education, University of Niš, Niš, Serbia; 2 National University of Physical Education and Sport, Kyiv, Ukraine; 3 Institute of Psychology, German Sport University Cologne, Am Sportpark Müngersdorf 6, 50933 Cologne, Germany; 4 Faculty of Kinesiology, University of Zagreb, Zagreb, Croatia

**Keywords:** Anatomy, Health occupations, Engineering

## Abstract

The aim of this study was to determine inter-instrument reliability between Takei and Jamar dynamometers in school children. Fifty-six five grade participants aged eleven to twelve (n = 32 boys, n = 24 girls) performed handgrip strength test on two different occasions, with a 5-day gap between them, as test–retest. The Pearson correlation coefficient showed very large to almost perfect correlation between both devices (r = 0.76–0.91) which was graphically confirmed by Bland–Altman method. Test–retest also showed high reliability (ICC = 0.78–0.85) for Jamar and Takei. Trivial, nonsignificant differences (*p* > 0.05) were observed between for test–retest trials for Takei left hand (ES = 0.04), right hand (ES = 0.12) and Jamar left hand (ES = 0.15). According to the results, both the Jamar and Takei dynamometers are valid and reliable for measuring schoolchildren, and both devices may be used to assess a student's handgrip strength for this age group.

## Introduction

Strength training has been shown to be a secure and efficient way to condition children, as long as the right exercise standards are followed^1^. Yet, reports show increases in some components of muscular fitness, but there are studies in children that shows declines in handgrip strength^2,3^. Low muscle strength is independently associated with a poorer metabolic profile during adolescence and with disease and all-cause mortality in adulthood^4^. Quantitative muscle strength measurement is indispensable in the evaluation of subjects, due to the monitoring of children's development^5^. Maximum voluntary handgrip strength (HGS) can assist researchers in identifying kids and teenagers at risk for serious public health issues such an increased chance of developing cardiovascular illnesses and poorer skeletal health in the future^6^. Considering the low levels of strength observed in children globally and the future health problems that may be developed consequently, HGS seems to be especially important to start measuring in children to track their trajectory as they develop^6,7^.

Two of the most discussed dynamometers within the current literature are the Jamar and the Takei^8^. The Takei dynamometer is characterized as a digital dynamometer, yet Jamar dynamometer is found to be a hydraulic tool^9,10^. The Jamar dynamometer is comprised of an adjustable anatomical rigid handle, hydraulic system, and analog display, which is regarded as the gold standard and frequently used as a benchmark in validity-reliability research, has a high test–retest reliability^11^. In grip strength, measurements conducted in the extended and 90° flexed positions of the elbow joint while standing, grip strength in elbow extension was found to be significantly higher than in elbow flexion^11^. Studies showed that Jamar hand dynamometer can be used in measuring children aged ten to twelve years^12,13^. Study carried out by Wołkow^14^ has demonstrated that children and adolescents have three distinct sensitive phases of the development of their motor abilities: between the ages of 8 and 9 years, 10 to 12 years, and 13 to 14 years. The research findings support this assertion by giving conflicting findings. A more balanced growth without abrupt accelerations or leaps was found to be a significant characteristic in the development of young judokas' motor abilities^15,16^. However, another research^17^ noticed an unusual trend in the rate of improvement in some motor abilities that was not seen in strength measures. After peaking between the ages of 11 and 12, the pace of development declines with age. The reliability of a Jamar dynamometer and the Martin Vigorimeter was compared for the measurement of grip strength in children^12^. As expected, smaller children needed a wider grip to use the Jamar dynamometer, but both can be used to measure children under twelve years old.

Similar to the Jamar, the Takei has also been found to be a valid and reliable tool to measure power grip. It uses an adjustable rectified and complacent handle shape, electromechanical system and a digital or analog display^8^. Several studies confirmed that Takei can be used to measure handgrip strength in children aged ten to fifteen years old^18–20^. We can see that Takei dynamometer is more used in measuring children, where Takei is used in a study involving 2125 children and adolescents^21^. Also, the preschool children aged 2–5 years old were tested with different dynamometers and observed that the Takei was the most reliable among the dynamometers, in addition, the Takei dynamometer showed the best validity^22^.

Considering previous studies, both dynamometers, Jamar and Takei, have shown validity and reliability in the child population^8,11^. Takei has already proven to be a valid instrument for measuring HGS in children and adolescents^18–20^. Also, Takei was constantly mentioned as the best, the most valid and most used, so it was necessary to check and compare the two dynanometers at school age. Considering the fact that Jamar is proven as the gold standard^11^, it is good to determine the inter-instrument reliability differences between these two devices and thereby confirm the fact about the validity of the device. Also, it is important to determine the validity of both devices, since it has already been confirmed that the Takei is easier to use on a child sample ^8^, since a smaller grip width is required, which can be a disturbing biological factor in testing children. For this reason, it is necessary to compare these two devices on a sample of school children aged 11–12 years. Although there is already research on this topic, this represents evidence-based data of the importance of using both dynometers Therefore, the aim of this study was to to examine inter-instrument reliability between Takei and Jamar dynamometers.

## Methods

### Participants

Fifty-six children (24 girls) aged from 11 to 13 years old (12.2 ± 0.4), from elementary school in Zagreb were tested (Table 1). Before testing, all participants were familiarized with the protocol and gave voluntary consent to participate in the study. Inclusion criteria considered healthy children that were attending school. For exclusion criteria kids that had current or recent injuries on their hands or previous hand surgery, that complaints of weakness or pain, were not included in this study. Prior to testing, written informed consent was obtained from the parents for each child. Additionally, children were asked if they participate in sports, if yes, in which sport, for how long and how many practices do they have weekly. This study was approved by the Committee on Ethics of Scientific Research at the Faculty of Kinesiology in Zagreb. We confirm that all methods were performed in accordance with the relevant guidelines and regulations of the Declaration of Helsinki. The study is part of the institutional project: The influence of wrestling training on selected tests of motor skills and body composition in Croatian wrestlers (reference number: 8835/2022).

**Table 1:** Basic descriptive characteristics.

Variables				
n	Age (years)	Body height (cm)	Body mass (kg)	BMI (kg·m^2^)
56	12.2 ± 0.4	151.3 ± 6.9	43.7 ± 10.8	18.9 ± 3.6

Values are expressed as mean ± SD.

*BMI* body mass index.

### Testing procedures

The measurements were conducted during a physical education class in the school hall, at an ideal temperature. Both measurements occurred around 1 p.m. The tests were conducted on two separate occasions, with a 5-day gap between them, and all the equipment, preparations, administration time, and processing methods remained consistent for both measurements. While searching the literature, we were unable to identify any material suggesting that there was a gold standard for the period between two handgrip strength tests. The information on the period between two testing sessions (test–retest) varied among the examined studies^11,23–25^. In line with studies that used a similar design and lasted a week, and due to the fact that school suggested the most suitable time for retest, we have chosen to have a 5-day period between testing sessions. Prior to the testing, a 5 min warm-up was provided, and the tests were demonstrated to the children. Following the protocol described below, the participants were given an opportunity to practice the tests. The handgrip test scores were recorded by a researcher who had extensive experience in conducting and evaluating physical fitness tests.

Each child was given a brief demonstration and verbal instructions for the handgrip test using the Takei handgrip dynamometer (Takei Scientific Instruments Co. Ltd, Tokyo, Japan), which, if necessary was adjusted according to the child’s hand size. The participants stood with their arms by their sides and their elbows fully extended during this. Two times of alternate measurements were made without a break. The participant was instructed to squeeze the dynamometer for 3 s for each measurement^8^. The peak value of strength was recorded, and the better result was used in the analysis.

The Jamar dynamometer (Paterson Medical, Green Bay, WI, USA) was placed in the right and left hand and was held loosely around the readout dial by the examiner to prevent dropping^26^. Two maximum contractions were taken for each hand. The better result of each hand was used for analysis. All participants were examined in the sitting position^27^.

### Statistical data processing

The central tendency and variability of the data were represented using means and standard deviations (SD), along with 90% confidence interval limits (90% CI). The normality of the data was assessed using the Shapiro–Wilk test. Paired sample t-tests were conducted to determine if there was a significant difference between the Jamar and Takei test–retest measurements for both hands. Standardized differences in means were calculated to assess the magnitude of the change within and between the tests. The effect size (ES) magnitudes of change were categorized as trivial (> 0.2), small (0.2–0.5), moderate (0.5–0.8), large (0.8–1.60), and very large (> 1.60)^28^. The reliability of the mean change between trials was evaluated using the intraclass correlation coefficient (ICC), typical error (TE) and coefficient of variation (CV%). ICC values of 0.1, 0.3, 0.5, 0.7, 0.9, and 1.0 were classified as low, moderate, high, very high, nearly perfect, and perfect, respectively. Good reliability was defined as a CV < 5% and ICC > 0.69^29^. The degree of association between Jamar and Takei measurements was assessed using Pearson's product-moment correlation (r). Correlation values were interpreted as indicating small (r = 0.1–0.3), moderate (r = 0.3–0.5), large (r = 0.5–0.7), very large (r = 0.7–0.9), and almost perfect (r = 0.9–1.0) associations between variables and tests. Statistical significance was indicated in cases where *p*-value was less than 0.05. Additionally, the agreement between Jamar and Takei dynamometers data was then examined graphically using Bland and Altman’s plots in which the difference between both devices was plotted against the mean of the two devices^30^.

## Results

### Reliability

Test–retest reliability statistics for outcomes measures attained from Jamar and Takei both left and right hands are displayed in Table 2. Trivial, nonsignificant differences (*p* > 0.05) between the test–retest trials for Takei left hand (ES = 0.04), right hand (ES = 0.12) and Jamar left hand (ES = 0.15) were observed. Jamar right hand showed small differences (ES = 0.24). Furthermore, Jamar left hand (CV = 1.07%, ICC = 0.79), right hand (CV = 1.07%, ICC = 0.85) possessed good reliability ratings across test–retest trials. Takei left hand (CV = 0.98%, ICC = 0.78), right hand (CV = 0.99%, ICC = 0.83) also showed good reliability ratings across test–retest trials. Absolute reliability (CV) was considered as acceptable.

**Table 2:** Test–retest reliability in both dynamometers.

	Jamar		Takei	
	Left hand	Right hand	Left hand	Right hand
ES	0.15 (trivial)	0.24 (small)	0.04 (trivial)	0.12 (trivial)
Diff (kg)	3.43	5.93	1.10	3.17
ICC (90%CI)	0.79 (0.67; 0.87)	0.85 (0.75; 0.90)	0.78 (0.65; 0.86)	0.83 (0.73; 0.89)
TE (kg)	1.93	1.92	2.17	2.13
CV %	1.07	1.07	0.98	0.99

*ES* effect size, *ICC* intraclass correlation coefficient, *TE* typical error of measurement, *CV* coefficient of variation, *CI* confidence intervals.

### Pearson correlation

The mean ± SD for each outcome measure taken from Jamar and Takei left and right hand is shown in Table 3. Pearson correlation coefficients demonstrating the criterion validity between the Jamar and Takei were very large to almost perfect for right hand T1 (r = 0.84), right hand T2 (r = 0.91), and left hand T1 (r = 0.76), left hand T2 (r = 0.84) (Table 2). Differences between outcome measures observed in left hand T1, right hand T1 and right hand T2 were trivial (from 1.5 to 4.25%; ES = from 0.06 to 0.16) while small effect size was found in left hand T2 (5.40%; ES = 0.22).

**Table 3:** Correlation and comparison of Jamar and Takei.

	Jamar	Takei	Diff	ES	*r* (90% CI)	Rating
Left hand T1	18.64 ± 4.07	19.22 ± 4.26	3.06	0.13 (trivial)	0.76* (0.60–0.86)	Very large
Left hand T2	18.01 ± 4.39	19.01 ± 4.94	5.40	0.22 (small)	0.84* (0.74–0.91)	Very large
Right hand T1	20.50 ± 4.89	20.81 ± 4.84	1.50	0.06 (trivial)	0.84* (0.72–0.91)	Very large
Right hand T2	19.32 ± 4.91	20.16 ± 5.34	4.25	0.16 (trivial)	0.91* (0.86–0.94)	Almost perfect

*T1* test, *T2* retest, *ES* effect size, *CI* confidence intervals, *r* correlation coefficient; *(*p* ≤ 0.05) statistically significant.

### The Bland–Altman method

**Figure 1:**
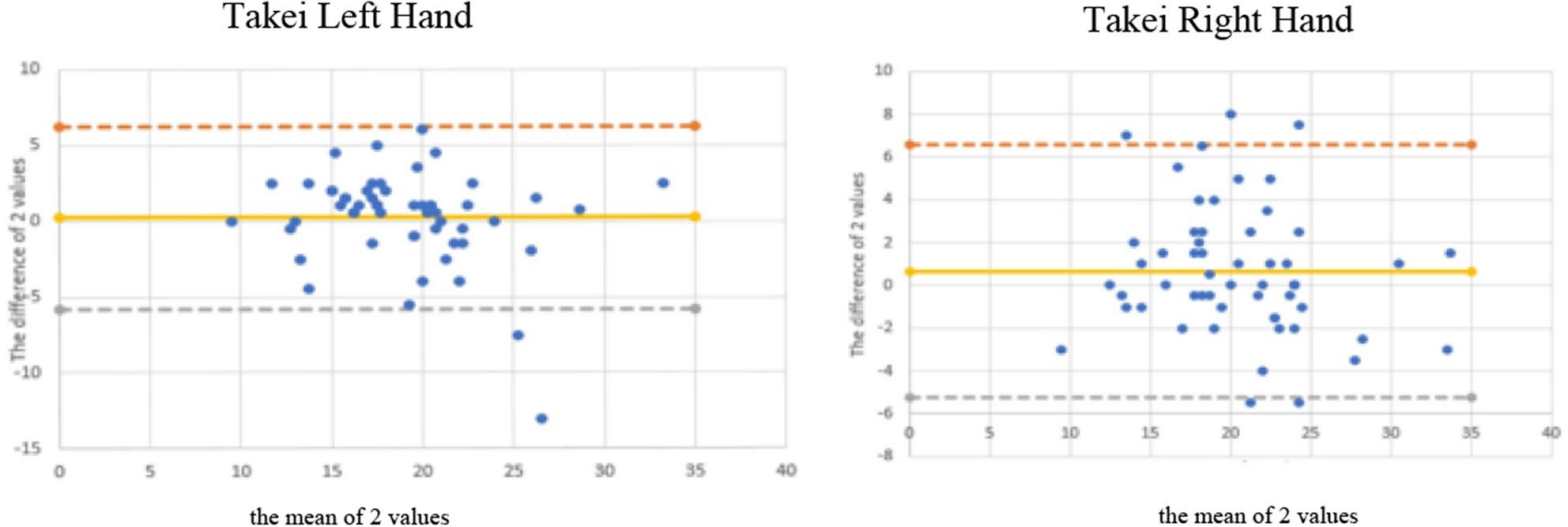
Bland–Altman plots of the handgrip strength test, for the left and right hand using Takei and Jamar dynamometer. The central line characterizes the mean difference between test and retest values (systematic bias); the upper and lower lines characterize the upper and lower 95% limits of agreement.

**Figure 2:**
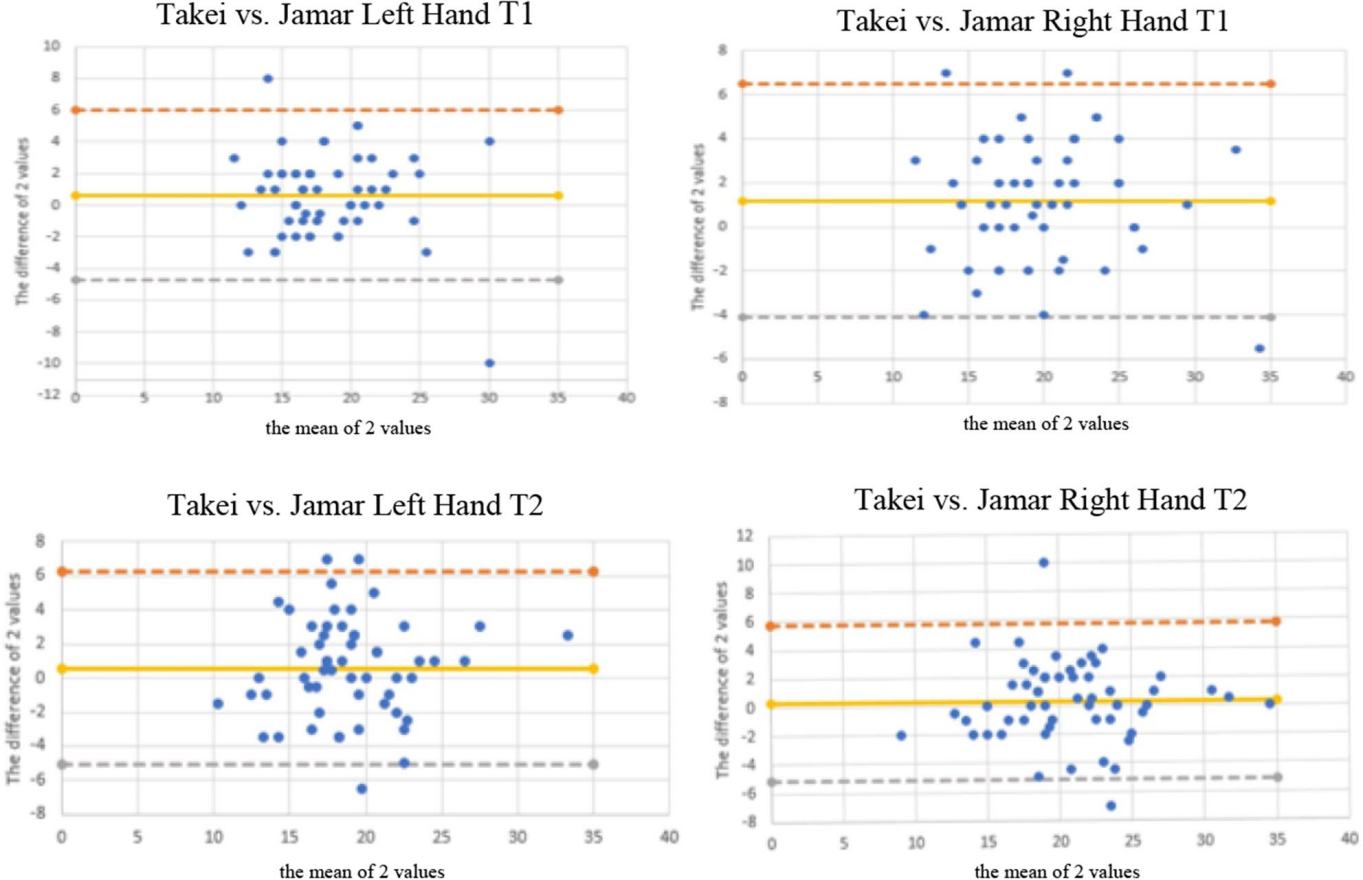
Bland–Altman plots of the handgrip strength test, between Takei and Jamar dynamometer for left and right hand on test (T1) and retest (T2). The central line characterizes the mean difference between test and retest values (systematic bias); the upper and lower lines characterize the upper and lower 95% limits of agreement.

The Bland–Altman method graphically showed the reliability patterns, and confirmed the results obtained from intraclass correlation in terms of systematic error (bias or mean intertrial differences) and random error (95% limits of agreement). This method also showed differences between the mean of test–retest of both hands grip strength using Jamar and Takei devices. Mean differences and most data points were within the limits of agreement (Figs. 1 and 2).

## Discussion

This study aimed to examine reliability of Jamar and Takei dynamometers in school children. The authors pointed out that younger and middle school age groups can demonstrate the best rate of development of motor abilities^31^. For this reason, they advise differentiating training at that specific time period in order to develop specific talents thoroughly while adhering to the basic requirements of the organism. The rate of biological growth has a major role in determining sensitive times. This makes it even more crucial to differentiate training methods appropriately^14^. Motor abilities, which have developed as a whole biological system with several subsystems as a result of the training, have significant interactions with one another. This kind of development is typical of the ontogenesis's pre-pubescence stage. The structure of motor abilities (namely, strength) changes significantly at the age of 12 years which makes it unique^17^. The main findings revealed that both dynamometers showed non-significant differences between test–retest trials performed with left and right hand. In addition, Jamar and Takei for both hands possessed good reliability ratings across test–retest trials. Absolute reliability was considered as acceptable. Pearson correlation coefficients demonstrated very large to almost perfect correlation between the Jamar and Takei dynamometers.

The results of our study (ICC = 0.79–0.85) are consistent with earlier research^12^ that found that handgrip strength in school children had strong test–retest reliability (ICC = 0.92–0.96) utilizing various hand dynamometer kinds and testing procedures. Our findings also collaborate with previous research^32^ where test–retest reliability was high for both preferred (ICC = 0.94–0.98) and non-preferred (ICC = 0.96–0.98). In accordance to obtained results are results of other study^33^ where intraclass correlation coefficient (ICC) showed correlation of 0.76–0.94. The present results showed high test–retest reliability values (ICC = 0.79–0.85) and the absence of significant differences between test and retest suggest that the familiarization process was sufficient to give the young athletes confidence in using the hand dynamometer and the isometric evaluation. Good to excellent test–retest reliability was shown for handgrip in school children (ICC = 0.90 and ICC = 0.94)^34^. In contrast to our study, Clerke et al.^35^ observed that adolescents (13–17 years old) had small but significant differences (*p* ≤ 0.05) in handgrip strength between tests. This divergence in results can be seen as of consequence of maturation and due to natural biological growth and increases in hand size. The Jamar and Takei dynamometers were shown to be valid by Pearson correlation coefficients, which ranged from very high to almost perfect correlation (r = 0.76–0.91) for left as well as right hand. The similar magnitude of correlation was observed in our study and other research^11^. This study showed very strong positive correlation between two dynamometers (r = 0.91–0.94). In contrast to our results are results from study^36^ which demonstrated very weak correlation (r = 0.07–0.08) using Pearson correlation coefficient.

The reliability of the handgrip strength assessment may be impacted by several variables, including the type of dynamometer (Jamar vs. Takei), the body posture (standing vs. seated), and the position of the elbow joint. The Jamar and Takei hand dynamometer were applied in this study to assess handgrip strength. Preschoolers were evaluated between the ages of two and five, using various dynamometers as part of their study, and they found that the Takei dynamometer had the highest reliability and validity^22^. Jamar dynamometer, which has been consider as the gold standard for measuring grip strength in both adults and children, is a commonly used tool^36^. Higher reliability of maximum handgrip strength may potentially result from different elbow position or body posture. In study^32^, it was concluded that sitting position, with elbow position at 90° is the most frequently testing protocol for the assessment of grip strength.

Moreover, our results indicated that Jamar and Takei dynamometers are both valid and reliable in measuring school children and that both devices can be included in the testing of student’s handgrip for this age group. There are some limitations and issues connected to our study. Results are limited to school children. It is uncertain whether these findings can be applied to different age groups or individuals with different levels of physical activity. The age of 11 marks a crucial phase in a child's development, characterized by significant biological changes and the onset of puberty. Therefore, physical activity becomes highly significant^17^. As a limitation is also that we excluded parameters that can affect the results, such as body mass (BM), body mass index (BMI) and fat-free mass (FFT), as potential predictors of changes in handgrip strength between two measurements (Supplementary File 1).

## Conclusion

Based on the results of present study, it can be concluded that Takai and Jamar dynamometers possessed good reliability ratings across test–retest trials, for both left and right hand in school children. Furthermore, these dynamometers were proved to be valid for assessment of handgrip strength. In order to measure handgrip strength in school children with normal development and who are not athletes, sports and health professionals can use this type of dynamometer as a reliable and valid measurement tool.

### Supplementary Information

Supplementary Information.

## Data Availability

All data generated or analysed during this study are included in this published article (and its supplementary information file).
